# MicroRNA Targeting to Modulate Tumor Microenvironment

**DOI:** 10.3389/fonc.2016.00003

**Published:** 2016-01-19

**Authors:** Praneeth R. Kuninty, Jonas Schnittert, Gert Storm, Jai Prakash

**Affiliations:** ^1^Targeted Therapeutics Section, Department of Biomaterials, Science and Technology, MIRA Institute for Biomedical Technology and Technical Medicine, University of Twente, Enschede, Netherlands; ^2^Department of Pharmaceutics, Utrecht University, Utrecht, Netherlands

**Keywords:** tumor microenvironment, tumor stroma, microRNA, gene delivery, cancer-associated fibroblasts, tumor-associated macrophages

## Abstract

Communication between stromal cells and tumor cells initiates tumor growth, angiogenesis, invasion, and metastasis. Stromal cells include cancer-associated fibroblasts, tumor-associated macrophages, pericytes, endothelial cells, and infiltrating immune cells. MicroRNAs (miRNAs) in the tumor microenvironment have emerged as key players involved in the development of cancer and its progression. miRNAs are small endogenous non-protein-coding RNAs that negatively regulate the expression of multiple target genes at post-transcriptional level and thereby control many cellular processes. In this review, we provide a comprehensive overview of miRNAs dysregulated in different stromal cells and their impact on the regulation of intercellular crosstalk in the tumor microenvironment. We also discuss the therapeutic significance potential of miRNAs to modulate the tumor microenvironment. Since miRNA delivery is quite challenging and the biggest hurdle for clinical translation of miRNA therapeutics, we review various non-viral miRNA delivery systems that can potentially be used for targeting miRNA to stromal cells within the tumor microenvironment.

## Introduction

The tumor microenvironment is composed of cancer cells and non-cancerous cells so-called stromal cells such as cancer-associated fibroblasts (CAFs), tumor-associated macrophages (TAMs), pericytes, endothelial cells, and infiltrating immune cells (1, 2). Over the last decade, it was well established that stromal cells promote tumor growth, angiogenesis, invasion, and metastasis (3, 4). These effects are observed in breast, pancreatic, liver, brain, ovarian, and prostate cancer. Evidence suggests that tumor cells recruit stromal cells by secreting chemokines and growth factors, which educate them to create a tumor-favoring microenvironment (4). The “educated” stromal cells, such as CAFs, endothelial cells, pericytes, TAMs, and other immune cells, interact with tumor cells as well as among themselves to stimulate tumor growth, metastasis, and development of resistance to chemotherapy (3, 4). Intervening into these interactions within the tumor microenvironment is an interesting strategy to develop novel therapies for cancer treatment.

MicroRNAs (miRNAs) are represented as a novel class of therapeutics, regulating multiple signaling pathways within the tumor microenvironment (5). miRNAs, a class of small (17–25 nt) endogenous non-coding RNAs, regulate gene expression at the post-transcriptional level and thereby control cellular processes such as differentiation, proliferation, and migration (6). miRNAs have the ability to regulate not only one but also hundreds of target genes simultaneously and thereby control multiple signaling pathways (7). Gene silencing occurs through imperfect/perfect complementary base pairing between a miRNA guide strand and the 3′ UTR region of the mRNA, which leads to translational repression or mRNA degradation (6, 8). During cancer initiation and progression, the expression levels of multiple miRNAs are aberrantly up- or downregulated, resulting in an imbalance of cellular pathways ultimately leading to the attainment of a pathological state. In this article, we highlight dysregulated miRNAs in different tumor stromal cells and their functions in the regulation of the multifaceted tumor microenvironment. The expression of miRNAs can be controlled by administering either miRNA inhibitors (antagomiR) or miRNA agonists (miR mimics). We summarize various miRNA delivery approaches that have been or can potentially be applied to deliver miRNA as therapeutics into stromal cells.

## MicroRNA in the Tumor Microenvironment

**Figure 1:**
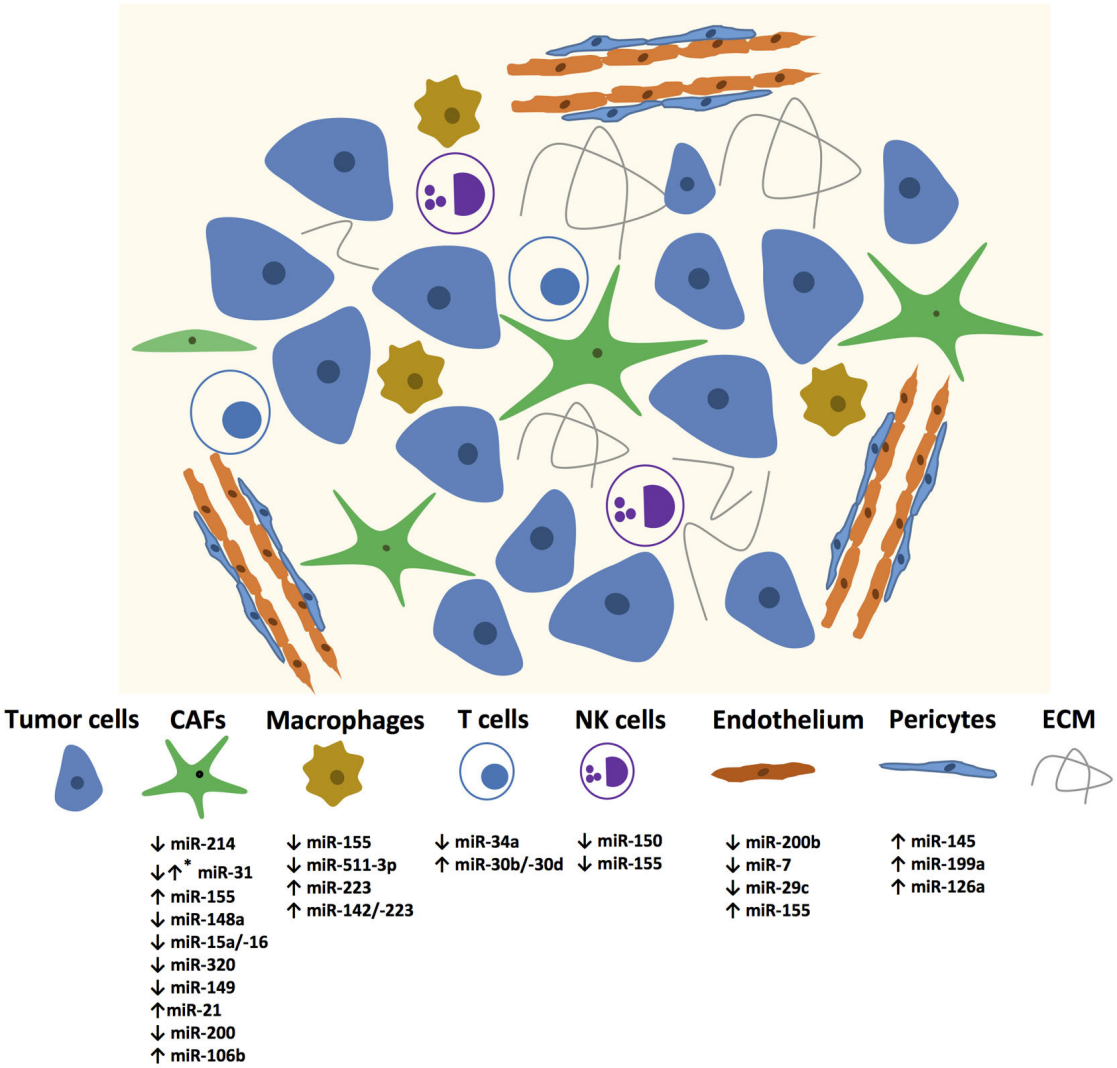
**miRNAs mediating changes in tumor microenvironment components**. Up and down regulated miRNAs are enlisted? *Mark denotes that the specific miRNA is expressed differentially in different CAFs.

In recent years, many miRNAs have been identified in different stromal cells of the tumor microenvironment as illustrated in Figure 1. The Table 1 summarizes the miRNAs in the stromal cells from different cancer types with genes regulated by these miRNAs and their functions.

**Table 1:** **List of miRNAs in various tumor stromal cells**.

Cell type	miRNA	Cancer type	Target gene	Functions
CAFs	miR-155/214 (9)	Ovarian	CCL5	Differentiation
	miR-31 (10)	Endometrial	SATB2	Migration, invasion
	miR-148a (11)	Endometrial	WNT10B	Migration
	miR-15/-16 (12)	Prostate	FGF2	Migration, proliferation
	miR-320 (13)	Breast	ETS2	Invasion, angiogenesis, tumor growth
	miR-106b (14)	Gastric	PTEN	Migration, invasion
	miR-200c (15)	Breast	Fli-1, TCF12	Migration, invasion
	miR-149 (16)	Gastric	IL-6	Differentiation, anti-stromal effects on tumor cells
	miR-21 (17)	Colorectal	RECK	Differentiation

Macrophages	miR-155 (18)	Hepatocellular carcinoma	C/EBPβ	Repolarization toward M2 phenotype
	miR-511-3p (19)	Macrophages expressing MRC1	ROCK2	Tumor growth, blood vessel morphology
	miR-26a (20)	Hepatocellular carcinoma	M-CSF	Recruitment of macrophages

T cells	miR-34a (21)	Hepatocellular carcinoma	CCL22	Infiltration of immune cells
	miR-30d (22)	Melanoma cells	GALNT7	Infiltration of Tregs cells

NK cells	miR-150, miR-155 (23, 24)	NK cells	SHIP1	Activation of NK cells

Endothelial cells	miR-200b (25)		ETS1	Angiogenesis, migration
	miR-29c (26)		IGF1	Angiogenesis, proliferation
	miR-7 (27)	Glioblastoma		Cell viability, migration, angiogenesis
	miR-155 (28)	Breast	VHL	Invasion, migration, proliferation, angiogenesis

Pericytes	miR-145, miR-126a, miR-199a (29)		Fli1	Migration

### Cancer-Associated Fibroblasts

Cancer-Associated Fibroblasts are one of the most abundant cell types in the tumor microenvironment of many solid tumors (30, 31). In response to inflammatory stimuli, quiescent fibroblasts differentiate into activated myofibroblast (CAFs), expressing increased levels of α-smooth muscle actin (α-SMA). CAFs secrete numerous cytokines, chemokines, and ECM components, which actively participate in tumor progression, invasion, and metastasis (32–35). Differentiation of fibroblasts into a CAF phenotype has been proposed to be regulated at the post-transcriptional level by miRNAs (36).

Several studies have reported the importance of specific miRNAs in the activation and transdifferentiation of fibroblasts to CAFs and CAF-induced tumorigenic actions (9–11, 37). miRNA-21 is one of the most common miRNAs that is reported to be induced in tumor cells and CAFs of pancreatic and colorectal tumors (17, 37–40). Inhibition of miR-21 using antagomiR reduced the migration/invasion of CAFs (38). Not only upregulation but also downregulation of certain miRNAs can induce a CAF phenotype. Tang et al. identified downregulated miR-200 as a direct regulator of reprograming fibroblasts into CAFs (15). Downregulation of miR-200 in normal fibroblasts accelerated their migration and invasion potential similar to CAFs (15). Bronisz and coworkers identified miR-320 as a downstream regulator of the PTEN (Phosphatase and tensin homolog deleted on chromosome 10) gene that controls cell proliferation and migration in CAFs and was co-expressed in tumor stroma of breast tissue. Loss of PTEN and miR-320 has shown to be involved in the reprograming of the tumor microenvironment to promote tumor invasion and angiogenesis (13).

Mitra et al. found downregulation of miR-31 and miR-214 and upregulation of miR-155 in their miRNA profiling, and reversal of the activities of these miRNA in these patient-derived CAFs reversed their phenotype (9). This study suggested that miRNA reprograms fibroblasts into CAFs, and, therefore, targeting of miRNA in stromal cells could be a therapeutic approach to treat cancer (9). In other studies, miR-31 and miR-148a were shown to be downregulated in endometrial CAFs (10, 11). Overexpression of miR-31 or miR-148a in these CAFs impaired their ability to stimulate migration and invasion of endometrial cancer cells, which were linked to the direct targets SATB2 and WNT10B for miR-31 and miR-148a, respectively (10, 11).

Using miRNA microarray analysis, several dysregulated miRNAs have been identified in breast CAFs, e.g., miR-31-3p, 221-5p, and 221-3p were upregulated and miR-205, miR-200b, miR-200c, miR-141, miR-101, miR-342-3p, let-7g, and miR-26b were downregulated (41, 42). Furthermore, many miRNA-responsive target genes and signaling pathways were revealed that regulate different cellular processes such as cell differentiation, adhesion, migration, proliferation, and cell–cell interaction (41). It is important to note that miR-31 was found to be downregulated in CAFs derived from ovarian and endometrial tumors (9, 10) while it was upregulated in CAFs from breast tumor (41), indicating that the same miRNA can have dual activities, thereby acting as an oncogene in one tissue and as a tumor suppressor in another.

In prostate CAFs, miR-15a and miR-16 were shown to be downregulated in CAFs obtained from 23 patients (12). Downregulation of miR-15 and miR-16 in CAFs promoted tumor progression through the reduced post-transcriptional repression of Fgf-2 and its receptor Fgfr1. These pathways act on both stromal and tumor cells to enhance cancer cell survival, proliferation, and migration (12). Reconstitution of tumor-suppressive miR-15a and miR-16 in CAFs inhibited tumor-promoting ability of stromal cells as shown in a co-injection (tumor cells and CAFs) mouse model, proposing these miRNA as potential targets for the development of novel therapies (12).

Additionally, Li et al. demonstrated that miR-149 mediates the crosstalk between the tumor cell and CAFs in gastric cancer via prostaglandin E2 (PGE2) and interleukin (IL)-6 signaling pathways (16). While it remains unclear how PGE-2 modulates this crosstalk, it was demonstrated that by targeting IL-6, miR-149 inhibited fibroblast activation (16). The effects of CAFs on gastric cancer development were negatively regulated by miR-149 (16). Additionally, CAFs enhanced the epithelial to mesenchymal transition (EMT) and stem-like properties of gastric cancer cells in a miR-149-/IL-6-dependent manner (16). In another study, miR-106b has been identified as a marker of poor prognosis in gastric cancer (14). Downregulation of miR-106b expression in CAFs resulted in significantly inhibited CAF-induced gastric cancer cell migration and invasion mediated through PTEN pathway (14). These studies reveal the miRNA targets as diagnostic biomarkers and therapeutic targets for developing the anti-CAF therapy.

### Tumor-Associated Macrophages and Immune Cells

Macrophages and other immune cells such as T-cells and natural killer (NK) cells are the major inflammatory cells infiltrating into the tumor microenvironment (3, 43). In the past, the infiltration of innate and adaptive immune cells into the tumor microenvironment was considered as an immune attack against cancer (43). However, now it is widely accepted that immune cells do also promote cancer initiation, progression, and metastasis (44). Macrophages within the tumor microenvironment can be polarized from antitumorigenic M1 macrophages to pro-tumorigenic M2 macrophages [TAMs, via changes in their metabolic pathways and the production of cytokines (CSF-1, IL-4, IL-13) by immune cells (43)]. TAMs promote tumor progression by stimulating angiogenesis, tumor cell migration, and extravasation at metastatic sites and suppressing antitumor immunity thereby reduce patient survival (43, 45, 46). Recent studies have unraveled the significance/dysregulation of miRNA in macrophages (47). In a study by Graff et al., miRNA expression profiles were determined in monocyte-derived macrophages differentially polarized into M1, M2a, M2b, and M2c phenotypes (48). They reveal several miRNAs to be uniquely regulated in human macrophages polarized into M1 (miR-125a-3p, miR-26a-2*), M2a (miR-193b), and M2b (miR-27a*, miR-29-b-1*, miR-132, miR-222*) (48). Herein, we report approaches through which dysregulated miRNAs have been targeted to reprogram miRNA expression in TAMs and thereby suppress their pro-tumorigenic properties.

Squadrito et al. showed that miR-511-3p, encoding for the macrophage mannose receptor, is upregulated in MRC1^+^ TAMs (19, 49). Enforcing miR-511-3p expression in MRC1^+^ TAMs resulted in the suppression of pro-tumoral genes and inhibited tumor growth with a change in blood vessel morphology. These effects were attributed to ROCK2, a direct target of miR-511-3p (19). The protein expression of transcription factor C/EBPβ showed elevated levels in TAMs as well as human hepatocellular carcinoma (HCC) tumor sections *in situ* (18). C/EBPβ expression was correlated with the production of cytokines in tumor-activated monocytes and shown to be regulated by sustained reduction of miR-155 (18). Overexpression of miRNA-155 was shown to attenuate the production of the cytokines (IL-6, IL-10, and TNF-α) by suppressing C/EBPβ expression, which led to inversion of pro-tumoral M2 into pro-inflammatory M1 macrophages, as demonstrated by upregulated M1 markers (TNF-α, NOS2, and IL-12) and downregulated M2 markers (Arg1, Ym1, Msr2, Fizz1, and IL-10) (50). More recently, ectopic expression of miR-26 in HCC cells suppressed the tumor growth, downregulated the expression of macrophage colony-stimulating factor (M-CSF) through the PI3K/Akt pathway, and suppressed the infiltration of macrophages in tumors (20). In addition, miR-26a expression was inversely correlated with M-CSF expression and the infiltration of macrophages into the tumor tissue of HCC patients (20).

Besides TAMs, other immune cells such as myeloid-derived suppressor cells (MDSCs), NK cells, and T cells also express miRNAs that regulate their pro-tumorigenic potential. MDSCs negatively regulate immune responses by suppressing the antitumor functions of CD4^+^ and CD8^+^ T cells by inhibiting the activities of NK cells (51). miR-155 and miR-21 are reported as the most upregulated miRNAs in MDSCs from bone marrow cells, regulating PTEN and SHIP1, respectively (52). They promote STAT3 activity by inducing MDSC expansion that promotes tumor aggressiveness via immunosuppression (52). In HCC, positive for the hepatitis B virus, suppressed levels of miR-34a resulted in the enhanced production of chemokine CCL22, thereby recruiting regulatory T cells (Tregs) into the tumor microenvironment to facilitate immune escape (21). In human melanoma upregulation of miR-30b/-30d correlates with stage, metastatic potential, shorter time to recurrence and reduced overall survival (22). Upregulation of miR-30d in the immunocompetent mice triggered immunosuppressive properties at the lung metastatic site, shown by an enhanced infiltration of Tregs (22). Bezman et al. suggested that miR-150 differentially controls the development of NK and invariant NK T (iNKT) cells by targeting c-Myb. Mice with miR-150 deletion showed a defect in their ability to develop mature NK cells (23). Overexpression of miR-150 promotes the development of mature NK-cells, which were highly responsive to activation (23). Contrarily, the number of iNKT cells was reduced upon miR-150 upregulation (23). MiR-155 was found to regulate partly interferon-gamma production in human NK-cells by downregulating SHIP1, making it a potential target in neoplastic disease (24).

### Tumor Vascular Cells

Endothelial cells together with pericytes are the major cellular components of tumor blood vessels, thus playing an important role in angiogenesis during tumor development (2–4). Chan et al. identified avian erythroblastosis virus E26 (v-ets) oncogene homolog-1 (Ets-1), an angiogenesis-related transcription factor, regulated by miR-200b (25). Ectopic expression of miR-200b reduced the tube formation and cell migration ability of human microvascular endothelial cells (HMECs) *in vitro* by targeting Ets-1 and its associated genes, matrix metalloproteinase-1 and VEGF receptor-2 (25). Interestingly, the authors demonstrated that miR-200b downregulation is hypoxia-induced and represses Ets-1 expression to promote angiogenesis in HMECs (25). Hypoxia stimulation also influences several miRNA expression levels in rat cortical pericytes compared to normoxic conditions (29). Real-time PCR data revealed changes in the expression of miRNAs associated with hypoxia-inducable factor-1α (HIF-1α) (miR-322 and miR-199a), TGF-β1 (miR-140, miR145, and miR-376b-3p), and VEGF (miR-126a, miR-297, miR-16, and miR-17-5p) (29).

In human umbilical vein endothelial cells (HUVECs), miR-29c was identified to regulate cell cycle, proliferation, and angiogenesis *in vitro*, likely mediated by suppressing Insulin-like growth factor-1 (26). miRNA-7 was identified as a negative regulator of angiogenesis, strongly reducing cell viability, tube formation, sprouting and migration *in vitro* (27). In an *in vivo* murine neuroblastoma tumor model, angiogenesis and tumor growth were significantly inhibited upon local administration of miR-7 (27). MiR-155, which is known to be upregulated in various human cancers, is also involved in the angiogenesis of breast cancer by targeting the von Hippel–Lindau (VHL) tumor suppressor gene (28). Mammary fat pad xenotransplantation of ectopically expressed miR-155 strongly induced angiogenesis, proliferation, tumor necrosis, and recruitment of pro-inflammatory TAMs (28). Moreover, miR-155 was identified as a marker for late-stage, lymph node metastasis and poor prognosis in breast cancer (28).

Recently, miRNA array profiling revealed that endothelial cells communicate with pericytes via miRNA-containing exosomes, which increased VEGF-B expression in pericytes at both gene and proteins levels (53). In addition, Lim et al showed that CXCL12-specific miRNAs are transported from bone marrow stroma to breast cancer cells via gap junctions and reduced CXCL12 levels as well as proliferation (54). Furthermore, mechanisms for the transfer of siRNA and miRNA through the gap junctions have been reviewed (55).

## MicroRNA Delivery to the Tumor Stromal Cells

To turn miRNAs into therapeutics, it is essential to deliver antagomiRs or miRNA mimics cell specifically into the target cells. However, naked miRNAs are unable to pass through cell membranes due to their hydrophilicity, polyanionic nature, and high molecular weight. Intravenous administration of naked miRNA leads to poor tissue distribution due to rapid renal excretion (plasma half-life ~1 h) and degradation by serum RNases (56). *In vivo* application of miRNA either requires a chemical modification or formulation into delivery systems. There are several articles describing targeting strategies for miRNA delivery (57–64). Despite having many strategies reported, the number of successful delivery approaches *in vivo* is still limited.

### Strategies for miRNA Delivery

Encapsulation of miRNAs into nanoparticles has been addressed to protect miRNAs from degradation by nucleases, resulting in an improved circulating half-life when administered systemically (65). Viral and non-viral encapsulation strategies have been investigated for this purpose. Viral-based delivery of nucleotides shows high transfection efficiencies, but will not be discussed in this review due to concerns regarding strong inflammatory side effects (66). The advantages of non-viral delivery systems over viral delivery systems are their well-defined molecular composition, simpler manufacturing and considerably lower immunogenicity (66). The advantages of non-viral delivery systems over viral delivery systems are their well-defined molecular composition, simpler manufacturing and considerably lower immunogenicity (67).

Non-viral delivery systems that have been investigated for their usability as miRNA carrier systems are liposomes, lipoplexes, and polyplexes. Liposomes are composed of phospholipids possessing a hydrophilic head linked to a hydrophobic tail (68). This amphiphilic structure allows them to form vesicles with an inner aqueous compartment in which miRNAs can be encapsulated (68). Cationic lipids have been used to form complexes with negatively charged miRNA, called lipoplexes (69). Liposomes and lipoplexes have shown to protect miRNAs from degradation by nucleases, resulting in an improved circulating half-life when administered systemically (65). In addition, cationic polymers that are frequently used for intracellular delivery are polyethyleneimine (PEI) and polyamide amine dendrimers (PAMAM). PEI- and PAMAM-based polyplexes have been used successfully for the delivery of miRNA and *in vitro* and *in vivo* (70, 71). Systemic delivery of PAMAM had toxic effects on the liver and the kidney in mice (72). For PEI, clinical translation has been hampered by dose-dependent toxicity upon systemic administration (73). Similar toxicity issues can be expected from lipoplexes.

### MicroRNA Delivery to Tumor Stroma Cells

The number of studies for the delivery of miRNAs to the compartments of the tumor stroma *in vivo* is highly limited. Polyplexes based on PEI delivered dicer substrate RNA duplexes, mimicking the structure of endogenous precursor miRNA-155 hairpin (Dmi155), into ID8-Defb29/Vegf-A tumors in mice (74). Increasing the levels of miRNA-155 in tumor-associated dendritic cells silenced multiple immunosuppressive mediators (74). These changes lead to the transformation of tumor-infiltrating dendritic cells from their immunosuppressive phenotype to immunostimulatory cells (74). Post-transformation of these cells were capable of triggering antitumor responses thereby inhibiting the progression of established ovarian cancers in mice (74).

Endothelial cells are the only cell type present in the tumor microenvironment, which have been actively targeted *in vivo* for miRNA delivery. Anti-angiogenic effects in tumors were achieved in a study performed by Ando et al. using lipoplexes modified with a PEG chain bearing the angiogenic vessel targeting peptide Ala–Pro–Arg–Pro–Gly (APRPG) (75). The APRPG–PEG-modified liposomes were used to form complexes with miR-499. The resulting lipoplexes were injected into mice bearing Colon 26 NL-17 xenografts (75). The lipoplexes accumulated in both angiogenic vessels and cancer cells resulting in downregulation of miR-499-regulated proteins and vascular endothelial growth factor (VEGF) (75). Following an injection of 0.5 mg/kg of the lipoplexes, tumor growth in mice was significantly inhibited (75).

In a study by Liu et al., anti-miR-296 antagomiR was delivered into HUVECs (76). PEGylated liposome-polycation-hyaluronic acid (LPH) nanoparticles conjugated with a cyclic RGD peptide (cRGD) were used as carrier systems for the specific targeting of α_v_β_3_, a receptor present on endothelial cells in the tumor neovasculature (76). They reported inhibition of tube formation and endothelial cell migration, and the significant upregulation of hepatocyte growth factor-regulated tyrosine kinase substrate (HGS), one of the genes suppressed by miRNA-296 (76). A matrigel plug assay was performed to analyze the effect of anti-miR-296 delivery on *in vivo* angiogenesis (76). As a result, a decrease in microvessel formation by preventing CD31-positive endothelial cells from invading into the matrigel in combination with an induction of HGS in angiogenic endothelial cells was observed (76).

Anand et al. aimed to repress neovascularization in tumors by inhibiting miRNA-132 levels via the delivery of anti-miRNA-132 (77). Liposomal nanoparticles composed of distearoylphosphatidylcholine (DSPC), cholesterol, dioleoylphosphatidylethanolamine (DOPE), distearoylphosphatidylethanolamine (DSPE)-mPEG2000 modified with a DSPE–cRGD, targeting α_v_β_3_ were used as a delivery system (77). miR-132, highly overexpressed in the endothelium of human tumors, mediated loss of p120RasGAP thereby inducing neovascularization (77). By restoring p120RasGAP levels using anti-miR-132 delivery, neovascularization was suppressed, and tumor burdens were decreased in an orthotopic xenograft mouse model of human breast carcinoma, and vessels were maintained in their non-pathological resting state (77). Systemically administered miRNA7 loaded peptide/polymer-based delivery systems modified with a cRGP ligand targeting the integrins α_v_β_3_ and α_v_β_5_ (78), targeted human glioblastoma xenografts in mice and strongly reduced angiogenesis and tumor proliferation.

## Conclusion

In recent years, miRNAs have been extensively discovered in the tumor stromal cells either to be used as biomarkers or to show their potential for inhibiting cellular processes. Undoubtedly, the miRNA field has a high potential to develop novel therapeutics against cancer; however, at the same time development of technologies to deliver miRNAs to specific cells are highly essential to utilize them for therapeutic purposes. With this review, we bring together the fields of tumor biology and miRNA delivery, which will surely benefit both biologists and technology developers.

## Author Contributions

JP designed the structure of the review, wrote, and edited the review article. PK and JS wrote the review article. GS read the article.

## Conflict of Interest Statement

The authors declare that the research was conducted in the absence of any commercial or financial relationships that could be construed as a potential conflict of interest.
